# Suicidality Calls to National Helpline After a Terror Attack and War

**DOI:** 10.1192/j.eurpsy.2025.339

**Published:** 2025-08-26

**Authors:** S. Blum, G. Zalsman

**Affiliations:** 1 Geha Mental Health Center, Petah Tikva, Israel

## Abstract

**Introduction:**

IMPORTANCE Changes in suicide rates after a nationwide trauma may be different from changes in psychiatric symptoms or general distress after such events. However, very few studies have examined short-term suicide-related reactions after such an event.

**Objectives:**

To evaluate the short-term outcome of the events in Israel on October 7, 2023, a large-scale terror attack and unfolding war, on changes in suicidality as reflected in percentages of suicide-related calls in relation to all calls to a national mental health first aid helpline, the Israeli Association for Emotional First Aid (ERAN).

**Methods:**

DESIGN, SETTING, AND PARTICIPANTS The data included all interactions via the various ERAN helpline services between January 1, 2022, and December 31, 2023.

EXPOSURES The October 7, 2023, terror attack on Israel.

MEAN OUTCOMES AND MEASURES Changes in the numbers of overall calls and suicide-related calls to the ERAN helpline using an interrupted time-series analysis.

**Results:**

RESULTS. Analysis indicated that overall calls increased significantly on October 7. However, the number of suicide-related calls in the 3 months before October 7 was 1,887, whereas 1,663 suicide-related calls were registered in the 3 months after. The percentage of suicide-related calls decreased significantly on October 7 and gradually increased in the following period.

**Conclusions:**

CONCLUSIONS AND RELEVANCE The findings suggest that although short-term emotional distress increased after national trauma, the percentage of suicide-related calls decreased. These results support previous studies suggesting that suicidality is not one of the immediate reactions to such traumas

**Disclosure of Interest:**

None Declared

